# Wellbeing and Complementary Therapies in Breast Cancer Peripheral Neuropathy Care: A Scoping Review Focused on Foot Health

**DOI:** 10.3390/cancers15072110

**Published:** 2023-03-31

**Authors:** Raquel Veiga-Seijo, Maria Eva Perez-Lopez, Uxia Fernandez-Lopez, Abian Mosquera-Fernandez, Rocio Seijo-Bestilleiro, Cristina Gonzalez-Martin

**Affiliations:** 1 Department of Health Sciences, Faculty of Nursing and Podiatry, Campus Esteiro, Universidade da Coruña, 15471 Ferrol, Spain; 2 Research Group in Nursing and Health Care, Instituto de Investigación Biomédica de A Coruña (INIBIC), Hospital Universitario de A Coruña (HUAC), Universidade da Coruña (UDC), Sergas, 15006 A Coruña, Spain; 3 Research Group in Rheumatology and Health (GIR-S), Faculty of Physiotherapy, Campus Oza, Universidade da Coruña (UDC), 15008 A Coruña, Spain; 4 Medical Oncology Department, Instituto de Investigación Biomédica de A Coruña (INIBIC), Hospital Universitario A Coruña (HUAC), Sergas, 15006 A Coruña, Spain

**Keywords:** chemotherapy-induced peripheral neuropathy, breast cancer, foot health, quality of life, complementary therapy, evidence-based practice, rehabilitation

## Abstract

**Simple Summary:**

Chemotherapy-induced peripheral neuropathy is highly relevant in breast cancer because of the symptoms it triggers and its corresponding negative impact on health status. The foot is the most affected part of the body by this adverse effect. However, little is known about its implications and the most relevant complementary therapies to deal with it. Hence, this scoping review aimed to summarize the impact of this side effect on quality of life and foot health of people with breast cancer as well as describe the main assessment strategies and complementary therapies that may be used in clinical practice. Subsequently, by summarizing these findings, the aim was to communicate the key gaps in current knowledge and provide directions for new research as well as contribute to developing evidence-based practice in this highlighted field.

**Abstract:**

Background: Chemotherapy-induced peripheral neuropathy is a multidimensional health problem. Up to now, little evidence has been found concerning its impact on quality of life and foot health. Evaluation tools and prevention and treatment strategies must be reported. This study aimed to map the literature on the impact of this side effect on the wellbeing and foot health of people with breast cancer and to describe their main assessment strategies and complementary therapies. Methods: A scoping review was carried out while following the PRISMA-ScR and Arksey and O’Malley guidelines. Different databases (Cochrane Plus, Scopus, Web of Science, and Pubmed) were used. A total of 221 results were identified. Sixteen articles were included. Results: The thematic analysis obtained the following categories: the impact of peripheral neuropathy on quality of life and foot health, complementary therapies as a path for new strategies, and the need for clinicians and researchers to get involved in researching this side effect. Conclusions: Peripheral neuropathy has a negative impact on people’s quality of life. Implications for foot health and maintaining an active and healthy lifestyle have not been previously reported. Complementary therapies are recommended by scientific evidence, highlighting exercise. However, there is a need to develop more research that will help to incorporate them into evidence-based practice.

## 1. Introduction

Cancer is a global health problem, which implies an essential public health challenge [1]. According to GLOBOCAN 2020 [2], there will be approximately 19.3 million new cases and 10.0 million deaths by the year 2040. Among the most prevalent tumors, breast cancer (11.7%) is the most diagnosed regardless of sex, with 2.2 million cases reported in 2020 [3]. It is the most common type of cancer among women, and it is estimated that 1 in 12 women will develop it throughout their lives, making it their leading cause of death. Mortality is higher in low- and middle-income countries [2,4,5], in people older than 75 years [6], and in people with different comorbidities such as diabetes mellitus [7].

Today, there are more and more therapies available to treat breast cancer, although chemotherapy remains a mainstay. The most widely used drugs are anthracyclines, taxanes, 5-fluorouracil or capecitabine, cyclophosphamide, and carboplatin. These can trigger side effects that affect the person’s quality of life (QoL) and disease process. Likewise, the increase in survival of people affected by breast cancer leads to the need to develop a survivor-centered focus to address the toxicity caused by these treatments [8,9]. Chemotherapy-induced peripheral neuropathy (CIPN) is a common side effect that considerably impacts people with breast cancer. It is a set of symptoms that trigger sensory and motor nerve damage [10], giving rise to numbness, pain, burning or tingling sensations, sensitivity to cold or heat, and weakness. Approximately 60% of people are expected to develop it after neurotoxic chemotherapy [11]. These symptoms usually disappear progressively when treatment is stopped; however, there may be cases in which they last for a while or even become permanent (between 30% and 60%) [12]. This makes CIPN a treatment priority.

In broad terms, CIPN can affect the feet and hands of people, causing difficulties with mobility and balance and limiting the ability to participate in activities of daily living adequately. For example, Ducic et al. [13], who aimed to study balance and postural control in the lower limbs, reported that CIPN presents implications for gait or other noteworthy issues such as falls. Despite this, little evidence has been found on the repercussions of CIPN on foot health and its subsequent consequences. Thus, in light of this multidimensional health problem, professionals must improve the evaluation and monitoring aspects of treatment as well as prevention and treatment strategies. What is more, another key aspect of CIPN’s severity is its implication in the reduction or suspension of chemotherapy. For this reason, assessment, prevention, and treatment tools are very pertinent and necessary.

However, no study has been conducted that encompasses how CIPN impacts foot health and QoL as well as the main assessment and complementary therapy tools for it. For these reasons, a scoping review was conducted to systematically map the research done in this area as well as to identify any existing gaps in knowledge. The following objectives were established: (a) to explore the scientific evidence on the impact of CIPN on QoL and foot health in people with breast cancer undergoing chemotherapy, (b) to identify and describe CIPN’s main evaluation strategies and complementary therapies, and (c) to identify gaps in the literature to assist in planning future research.

## 2. Materials and Methods

A scoping review was conducted while following the extension of the Preferred Reporting Items for Systematic Reviews and Meta-Analyses Extension for Scoping Reviews (PRISMA-ScR) [14] guidelines for scoping reviews and by using the framework of Arksey and O’Malley [15] (October 2022). This paper aims to illustrate the starting point that will allow synthesizing knowledge from which future research priorities can be identified in the context of CIPN. Taking this into account, this document tries to reflect the main knowledge gaps about this field of study and the research needs that arise in clinical and experimental practice.

### 2.1. Identify the Research Questions

The research questions for this scoping review were the following: (1) What is known about the impact of CIPN on the QoL and foot health of people with breast cancer? (2) What is the nature of the scientific evidence on the primary assessment and monitoring strategies for CIPN and its most important complementary therapies?

### 2.2. Identifying Relevant Studies

The search was carried out in the Cochrane, Scopus, Web of Science, and Pubmed databases. This study implicated a systematic search with the following keywords: “peripheral neuropathy” AND “breast cancer” AND “chemotherapy” AND “foot”. The search strategy involved the following criteria: original articles, reviews, and conference papers published in English, Portuguese, and Spanish in the last 10 years. Publications were only included in the analysis if they addressed the problem of CIPN concerning the objectives and research questions previously quoted.

### 2.3. Study Selection, Charting the Data, and Analysis

A total of 256 potential results were obtained. After duplicates were removed, 221 citations were identified in electronic databases. Applying the previously referenced inclusion criteria and according to the variables in Table 1, the results were evaluated for inclusion in this research. Based on the title and the abstract, 185 were excluded because they focused on the effectiveness of targeted pharmacological treatment for the oncological process, they were focused on other types of cancers, and/or they were not based on chemotherapy agents. Thus, 36 full-text articles were retrieved and assessed for eligibility. After reading the 36 full-text papers, the authors excluded 20 articles. Of these, 6 were excluded because they did not focus on people with breast cancer, 13 did not address the questions raised in this research, and 1 was focused on pharmacological therapies.

**Figure 1:**
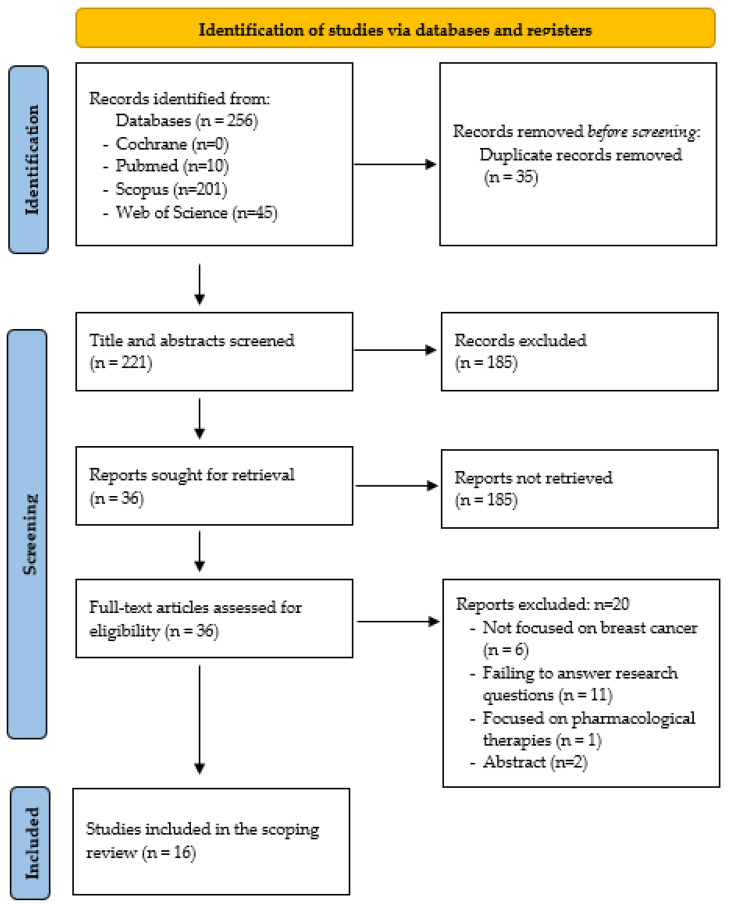
PRISMA flow diagram showing the studies included and excluded [14].

The authors reviewed and discussed each article to reach an agreement before making a final decision. Figure 1 shows the PRISMA flow diagram that outlines the identification and selection process of the documents included.

A descriptive analysis of the bibliometric parameters and a thematic analysis considering the aims of this review were carried out [14,15].

## 3. Results

A total of 16 studies were included in this scoping review. Table 2 shows a summary of the results obtained. What stands out in the table are the main characteristics of each article and the CIPN assessment tools and complementary therapies studied. Likewise, this section shows the bibliometric characteristics and the thematic categories that emerged from the thematic analysis. For this, the objectives of this review were considered.

### 3.1. Bibliometric Characteristics

#### 3.1.1. General Characteristics of the Documents Included

Most of the articles studied were published in the last 3 years (*n* = 9) [16,17,18,19,20,21,22,23,24]. Six documents were published in the United States [18,25,26,27,28,29], and two were published in Turkey [17,21]. No authors published more than one article. Concerning the type of work, four are pilot trials [17,23,25,29], and no document is a review article. Further to this, 12 papers are based on trials comparing two interventions or preventive measures for CIPN [17,18,20,21,22,23,24,25,26,27,29,30,31].

It is important to note that five papers were published in the *Supportive Care in Cancer* journal and three in *Breast Cancer Research and Treatment*. The rest of the papers were published in breast- or cancer-related journals.

#### 3.1.2. Study Population, Chemotherapeutic Agents, and Assessment of Neuropathy

The majority of the studies focused only on people with breast cancer (*n* = 9) [18,19,20,21,25,26,28,30,31]. The rest (*n* = 7) included samples with this type of tumor and people with other types of tumors. The most common chemotherapeutic agents were paclitaxel (*n* = 10) [18,19,20,21,25,26,27,28,30,31] and paclitaxel and platinum (*n* = 6) [16,17,22,23,27,29]. Only one article [24] included a wide variety of chemotherapy agents.

Concerning assessment tools, different scales and variables were used to evaluate CIPN, and no studies had this field as a study objective. The most widely used scales to evaluate CIPN were the National Cancer Institute Common Terminology Criteria for Adverse Events [16,17,18,20,23] (*n* = 5) and the European Organization for Research and Treatment of Cancer Quality of Life Questionnaire—Chemotherapy-Induced Peripheral Neuropathy, EORTC QLQ CIPN [19,22,23,25,30] (*n* = 5). Other scales used were the Total Neuropathy Score-Clinical Version [16,22] and the EuroQol5 Dimension 5 Level (EQ-5D-5L) [24]. Only one paper [31] used a questionnaire that included the person’s experience (Patient Neuropathy Questionnaire (PNQ)). They also assessed tactile disturbance using monofilament, thermosensory disturbance with a thermal stimulator, and vibration perception using a tuning fork. Griffiths et al. [26] also used monofilament as a complementary method for the assessment of neuropathy.

### 3.2. Thematic Categories

The thematic analysis allowed us to conclude the thematic categories presented below.

#### 3.2.1. Impact of Peripheral Neuropathy on Quality of Life and the Development of Comorbidities

Only one recent investigation (2022) [19] specifically studied CIPN’s impact on QoL. This article suggested that people who experience this side effect have worse overall QoL, functioning, and personal finances than those who are unaffected. Other scholars [16,24,28] studied different aspects related to CIPN and QoL, but this was not the main focus of their articles. Thus, the general study carried out by Hirose et al. [24] showed that adverse events such as peripheral neuropathy, general malaise, edema of the extremities, and dry skin are significantly correlated with a decrease in QoL. Wang et al. [16] indicated that the total score of The Functional Assessment of Cancer Therapy was significantly correlated with the scores of physical (*r_s_* = 0.54, unadjusted *p* < 0.001; adjusted *p* < 0.008) and functional (*r_s_* = 0.31, unadjusted *p* = 0.003; adjusted *p* < 0.008) wellbeing subscales. In addition, Bao et al. [28] reported that CIPN was associated with greater insomnia, anxiety, and depression (*p* < 0.05).

Another critical aspect to consider in relation to QoL and CIPN was analyzed by Simsek and Demir [21] and Griffiths et al. [26]. What stands out in their results is the need to monitor the symptoms and develop effective strategies as a key way to contribute to people’s QoL.

#### 3.2.2. Foot Health Repercussions: Gait and Active Lifestyle Issues

CIPN repercussions on foot health and QoL were not covered in the literature reviewed. One current published work (2022) [19] included results concerning foot health issues. They used the EORTC QLQ CIPN20 scale, which includes questions associated with foot symptoms and difficulty walking or standing. They showed that foot cramps and difficulty distinguishing between hot and cold water had a significant negative effect on QoL. Other aspects highlighted in this investigation were “numbness in the toes”, “difficulty climbing stairs or getting up from a chair due to weak legs”, and “trouble standing or walking due to difficulty feeling the ground underfoot”. In addition, they reported that after the illness, 4.7% declared having been on sick leave due to neuropathy. Survivors who registered moderate to severe “tingling in the toes/feet” (7%), “numbness in the toes/feet” (8%), and/or “foot cramps” (7%) would have preferred no treatment compared to the 3% who reported none or some of these symptoms (*p* < 0.05). Clinically important impairment in QoL was most prominent for “numbness in the toes and feet” and “difficulty walking because of foot drop” for all functional and personal finance scales.

Other works should be referenced because they bring to light different issues that may be relevant to foot health. For instance, Muller et al. [22] found that CIPN negatively impacts postural control and is associated with falls. Another recent publication [17] found that the feet and toes were more affected, as the top three reported problems were tingling (*p* = 0.003), numbness (*p* = 0.0001), and pain (*p* = 0.02).

#### 3.2.3. Complementary Therapies: The Focus of New Strategies for Peripheral Neuropathy

This section presents different complementary therapies that current research proposes as preventive or intervention measures for CIPN. The main therapies studied were cryotherapy (*n* = 6) [18,20,23,25,26,31], exercise programs (*n* = 3) [22,27,30], the application of cold and exercise (*n* = 1) [21], non-invasive skin electrostimulation (*n* = 1) [29], and the application of salt water (*n* = 1) [17]. Of the 12 documents, 4 had an intervention intention, and the rest (*n* = 8) had a preventive purpose.

The most researched complementary therapy was cryotherapy, which was applied differently in each study. Jue et al. [18] compared cold therapy with traditional care. People receiving traditional care were three times more likely to develop CIPN with progression toward severe neuropathy compared to cold therapy. They reported that this therapy is low-cost and -risk, and that it must be individualized due to differences in each person’s tolerance to cold. Likewise, Oneda et al. [23] observed good effectiveness and tolerability, as no patients had grade >2 CIPN. Ruddy et al. [25] used ice packs without obtaining any relevant results or changes in the scores of neuropathy scales (*p* = 0.26). Griffiths et al. [26] used gloves and socks. They indicated that only 24% of the participants were able to complete the study due to poor tolerance. In addition, they did not obtain significant differences in neuropathy and pain between the use or disuse of this therapy (feet *p*  >  0.30). Hanai et al. [31] used the same system without finding problems with cold tolerability. Concerning feet, the PNQ showed statistically significant differences between both groups (2.8% (intervention) vs. 36.1% (control), OR = infinite, 95% CI = 2.78 to infinite, *p* < 0.001).

A study that compared hilotherapy with frozen gloves [20] reported that hilotherapy was a better preventive measure concerning CIPN and nail toxicity. Hilotherapy resulted in greater prevention efficacy from a clinical and user perspective due to the comfort provided (*p* < 0.0001).

Exercise was the second-most widely studied type of therapy. Bland et al. [30] developed an aerobic and resistance exercise program that included five specific exercises that used machines, free weights, or resistance bands and involved performing repetitions depending on the type of exercise. There was a significant difference between groups in patient-reported moderate to severe numbness in the toes or feet (*p* = 0.04) and impaired vibration sense in the feet (*p* < 0.01). Similarly, Kleckner et al. [27] reported that exercise reduced CIPN-related symptoms of heat/coldness in hands/feet (*p* = 0.045) as well as numbness and tingling (*p* = 0.061). More statistically significant differences were found in men (*p* = 0.028), older people (*p* = 0.086), and people who had breast cancer (*p* = 0.076). In this line of therapy, Muller et al. [22] reported that subjectively perceived sensory symptoms in the feet increased less during chemotherapy in the adherent exercises than in the usual care group (*p* = 0.039).

Only one study [21] analyzed the comparison of cold application and exercise. Both interventions began after the first symptom and continued for 12 weeks. They found that exercise reduced symptoms of numbness in the hands (*p* = 0.009) and feet (*p* = 0.005) significantly compared to cold application and the control group.

One study [29] used Scrambler therapy and obtained a 53% reduction in pain, a 44% reduction in tingling, and a 37% reduction in numbness.

The latest complementary therapy investigated was salt water [17]. They found that cold salt water increased the general wellbeing of the individuals and improved their function and symptoms compared to warm salt water (*p* = 0.001).

#### 3.2.4. Need for Clinicians and Researchers to Become Involved in This Secondary Effect

The need to get involved in CIPN and investigate therapies for its prevention in depth was a prominent category theme. Wang et al. [16] point to the need for health professionals to get involved and pay attention to the needs of people who present this side effect in a holistic way and from multiple aspects. They promote considering people’s needs as the center of the approach to therapy.

Another field that must be addressed is that users need to receive adequate communication and information about the benefits and risks of CIPN. Engyall et al. [19] reported that most participating people would have refrained from treatment if they had known the consequences. Simsek and Demir [21] also encouraged informing and guiding people about the advantages and disadvantages of treatment.

Finally, other scholars [17,18,27] indicate that it is necessary to make professionals aware of complementary therapies for their use and offer them to people. Additionally, most of the studies emphasized the need to develop new strategies [25,26] or improve the therapies’ studies up to now. New strategies should focus on comorbidities, mental and emotional health, and fall prevention for aging breast cancer survivors [28].

## 4. Discussion

CIPN plays a critical role among the main adverse effects of chemotherapy, and it gained considerable importance in the scientific literature in recent years [26]. It is particularly meaningful in people with breast cancer because taxane and platinum therapy, the main choice in this type of cancer [32], is notable for triggering CIPN in a significant percentage of patients (during and after treatment). Despite this, very little literature was found on the implications of this adverse effect on QoL, standing, and gait and how complementary therapies could contribute to its prevention or reduction. This is especially important because CIPN has implications on QoL and the evolution of the oncological process. Therefore, it can lead to receiving inadequate doses of chemotherapy treatment (dose reduction, limitation, or even total cancellation) and modification of the drugs that people can receive for their disease [26,30].

Another significant aspect that has already drawn attention to the gap in the literature is that most of the studies belonged to pilot studies and were published in the last three years. To date, research tended to focus on the incidence and development mechanisms, etiology, and pathophysiological factors of CIPN [33,34].

### 4.1. Quality of Life, Foot Health, and Gait Implications

A strong relationship between CIPN, QoL, and breast cancer was demonstrated. Scientific evidence shows that CIPN impairs people’s QoL who experience it because it affects physical functions, activities of daily living, social and individual relationships, and all spheres of a person’s life [17,35]. Surprisingly, only one study (2022) [19] directly addressed CIPN and QoL, even though it can lead to deficient doses of potentially necessary treatment.

On the other hand, no study has comprehensively focused on the impact of CIPN repercussions on foot health. These implications are of considerable importance because CIPN affects mainly the extremities, which are essential for maintaining an active and healthy life. However, many investigations used CIPN rating scales that include items related to the foot [17,19]. In these studies, the foot symptoms were highlighted with relevant consequences, which indicates the need to carry out more investigations that consider this field specifically. Another significant aspect that has not been yet studied is to verify if some consequences of CIPN, such as falls, could also have etiology in the foot. This is another example of the need to approach this problem holistically, studying more aspects related to the foot.

### 4.2. Assessment Protocol

The next question in this study was about CIPN assessment tools. Surprisingly, no study focused on describing or delving into assessment techniques for this side effect. Despite this, the scales used in the included investigations were explored and collected. The variability in their use at a practical and research level was noticed; the literature is variable about the assessment methods usually used. Likewise, the EORTC, QLQ CIPN, and CTCAE were the most used in most of the included studies.

On the other hand, it is noteworthy that only two investigations employed monofilament for the assessment [26,31], although sensitivity problems are common in CIPN. Concerning the sensory changes in CIPN, a publication from 2022 [36] noted that there are still no methods available for early detection or specific biomarkers. Finally, only one study collected variables from the person’s perspective, when currently [37] it is recommended to include the subjective experiences of people with the evaluation of clinical professionals. This is relevant because research shows discrepancies between the results from both assessments. This knowledge is needed to inform different strategies better.

### 4.3. Complementary Therapies

Finally, it is worth mentioning that the complementary therapies collected in this review constitute an emerging and relevant topic in this field because an increase in the number of publications per year up to the present was observed.

First, concerning pharmacological strategies, several drugs have been tested, but effective strategies are lacking. In addition, these drugs only focus on CIPN sensory symptoms. Their efficacy varied among tests, although duloxetine and pregabalin were promising. The heterogeneity of their pathogenetic mechanisms may be a major issue hampering effective pharmacological strategies [38,39]. In addition, in most cases, these agents and more invasive modalities [21] may produce other unwanted effects [38,39]. These results suggest that the pharmacological strategies for CIPN are very limited.

Therefore, improving the symptoms experienced by people suffering from CIPN [38] requires multiple approaches adapted to the individuals [39], with a direction in methods that accompany each person and away from new and undesired adverse effects [40]. For this reason, it is necessary to approach complementary therapies that contribute to their wellbeing. Moreover, the Society for Integrative Oncology indicates that complementary and integrative therapies used as supportive care during cancer treatment are widely used by people with breast cancer [41].

The results of this work allow us to see that cryotherapy was one of the most used therapies in the included investigations. However, the most recently published trials are beginning to focus more on exercise than on cryotherapy. This may be because great benefits have not been observed in the use of cryotherapy, which is mainly due to the discomfort associated with the intervention [26]. Only two investigations of the six previously referenced obtained positive results (Jue et al. [18] compared it to traditional care, and Hanai et al. [31] used shorter administration times than the rest of the studies, which could increase tolerability). Likewise, there is significant variability in its use, as observed in the results described. Regarding exercise, it was effective for both prevention and treatment in all three articles studied. Although these studies also followed different methodologies regarding time and specific exercises, they were mainly based on aerobic exercise and strengthening methods. So far, only one study [21], which compared exercise and cryotherapy, found that exercise is more effective. Therefore, the summarized evidence shows us that exercise is the complementary therapy that seems the most effective in the latest research.

To conclude this section, the literature identifies other complementary therapies that were not considered because they did not include a sample with breast cancer. Once again, rehabilitation, strengthening programs, balance exercises, and non-pharmacological interventions stand out (endorsed by B recommendation levels [42]).

### 4.4. Implications for Clinical Practice and Future Research

Overall, this scoping review’s main practical implication is to raise awareness of the impact of CIPN comorbidities on the QoL of people with breast cancer. Another important practical implication is to improve the management of CIPN, which is a challenge faced by researchers and clinicians. Its care is relatively overlooked by health professionals compared to other adverse effects [43], despite its relevance in the literature.

The previous implications are supported by different studies. Kaley and DeAngelis [44] described that people might not report their CIPN symptoms because they fear missing out on effective cancer treatment. In this sense, the literature identified nurses as the reference professional who can best help them to understand information regarding treatments and their adverse effects [43,45]. Despite this, all healthcare professionals who monitor people with cancer and CIPN are called upon to get involved. Given the state of the science concerning the stated objectives, there are several implications and considerations for future research:

(a) The study of the implications of CIPN on foot health and an active lifestyle, as there is a gap in knowledge about this field.
(b) Agreeing on the CIPN assessment methods so the results can be comparable in terms of effectiveness between studies. Standardizing clinical assessment and creating procedural checklists will be necessary to ensure greater accuracy, as helpful diagnostic and assessment tools are lacking.
(c) Developing, deepening, and confirming safe and effective preventive strategies that can take place in oncology centers. Taken together, these findings do not support strong recommendations to validate a complementary therapy that healthcare professionals should recommend. Further research could also be conducted to determine the effectiveness of exercise [30]. Likewise, few studies aimed to compare therapies with each other [21]. Lastly, one study found that a range of activities can be effective for the self-care of CIPN symptoms in the feet, but more research is needed [46].

Overall, future projects must also include the following: better study methodologies and larger randomized controlled trials, which could provide more definitive evidence and consider different types of cancer, more geographic locations, more ethnic groups, and more chemotherapy treatments.

Finally, it is noteworthy that although the included studies describe walking and exercises in which the foot is involved, none of them approached the issue from the study of podiatry or the state of foot health to perform these interventions. This is relevant because many people can develop other problems, such as nail toxicity. These adverse effects may affect the adequate development of complementary therapies for CIPN, such as exercise. The evidence reviewed here seems to suggest a pertinent role for foot health-specialized professionals (podiatrists) working in a multidisciplinary team with other health care professionals (nurses, doctors, physiotherapist, occupational therapists, and so on).

Considering the aforementioned future research proposals, the ultimate aim is to be able to advise and apply complementary therapies in evidence-based practice to prevent or improve the symptoms of CIPN, which should be addressed explicitly in international clinical practice guidelines for breast cancer.

## 5. Conclusions

This scoping review highlights the impact of CIPN on the physical, functional, psychological, and emotional aspects of people’s QoL. The findings suggest that assessment methods have little consensus in the scientific literature. In addition, the present study is unique due to specifically reviewing CIPN’s implications on the foot and gait, which is the main part of the body affected. The importance lies in maintaining QoL and an active lifestyle in people with cancer receiving chemotherapy as well as avoiding more impacts on the oncological process.

This study identified key factors to contribute to the existing knowledge–practice gap in complementary therapies. Healthcare professionals should consider it in the clinical practice to contribute to patients’ QoL, as monitoring the symptoms of CIPN is a fundamental requirement to contribute to wellbeing. Exercise is the complementary therapy that seems to be the most effective in the latest research.

This booming topic invites further research, especially regarding foot health implications and exercise. For this purpose, more rigorous methodological designs are needed to inform clinical practice and guide health professionals about therapies based on scientific evidence to improve and establish evidence-based care in this field.

## Figures and Tables

**Table 1:** Inclusion criteria.

Variable	Inclusion Criteria
Year	Last 10 years
Study type	Original articles (randomized controlled trials, controlled clinical trials, case series, case reports, pilot clinical studies, longitudinal studies, and observational studies), reviews, and conference papers
Language	English, Portuguese, and Spanish
Population	>18 years
Cancer type	Breast cancer
Drug therapy	Chemotherapy
Scoping objective(s)	- CIPN repercussions on wellbeing and foot health - Main methods to assess CIPN - Main strategies to prevent or treat CIPN

CIPN: Chemotherapy-induced peripheral neuropathy.

**Table 2:** Quality of life, assessment, and complementary therapies for CIPN in people with breast cancer.

Author, Year, Country	Journal	Type of Study	Sample	Chemotherapy	Research Aim	Main Results	Assessment/ Complementary Therapy
Wang et al. [16] (2022), Hong Kong	Supportive Care in Cancer	Cross-sectional study	*n* = 87 Breast (42.5%) and colorectal (34.5%) cancer	Taxane, platinum	To identify the phenotype of CIPN and to examine the association with general symptoms and QoL.	Sensory symptoms are the most relevant. Worsening neuropathy contributes to a deteriorating QoL. It is associated with increased symptom burden, decreased physical wellbeing, and impaired tendon reflex.	*Assessment* NCI-CTCAE, TNSc, BPI, FACT/GOG-NtxS
Emine et al. [17] (2022), Turkey	Explore	Prospective experimental pilot study	*n* = 103 Breast (46.6%), colon (11.6%), gynecological (28.1%), and other (13.6%) cancers	Taxane, platinum, Taxane- platinum	To determine the effect of bathing with salt water in the management of CIPN.	Baths with cold salt water statistically significantly decrease the scores associated with the symptoms of peripheral neuropathy developed by taxanes and platinum. It is an effective approach to improve QoL.	*Assessment* NCI-CTCAE *Therapy*: Intervention Salt-water bath Temperature: warm group: 41 °C; cold group: 23–26 °C. Time: 30 min, every other day for two weeks.
Jue et al. [18] (2022), United States	Clinical Journal of Oncology Nursing	Randomized controlled trial	*n* = 48 Breast cancer	Taxane	To examine the difference in the severity and frequency of CIPN and QoL between the group receiving cold therapy and those receiving standard care.	Cold therapy effectively reduces peripheral neuropathy caused by taxanes. Regarding QoL, both groups had no significant differences.	*Assessment* FACT-Taxane questionnaire, CTCAE *Therapy*: Preventive Cold Therapy Temperature: −20 °C to −24 °C. Time: 15 min before and for one hour during treatment.
Engvall et al. [19] (2022), Sweden	Breast Cancer Research and Treatment	Cohort study	*n* = 646 Breast cancer	Taxane	To explore the impact of CIPN on QoL.	CIPN triggers clinically relevant deterioration in global QoL, functioning, and economics. The domains “Difficulty walking due to foot drop” and “problems standing/walking due to difficulty feeling the ground underfoot” are especially highlighted.	*Assessment* EORTC QLQ-C30, CIPN20
Coolbrandt et al. [20] (2022), Belgium	Breast Cancer Research and Treatment	Prospective self-controlled study	*n* = 63 Breast cancer	Taxane	To explore the efficacy of thread therapy on the right hand and foot and frozen gloves on the left hand and foot in people with breast cancer treated with paclitaxel or docetaxel.	Hilotherapy is a better alternative to prevent clinically significant taxane-related side effects compared to frozen gloves. Perceived comfort was statistically significantly better for hilotherapy (<0.0001).	*Assessment* NCI-CTCAE *Therapy*: Preventive Hilotherapy vs. frozen gloves Temperature: hilotherapy 10–12 °C; frozen gloves −18 to −20 °C. Time: hilotherapy: 120 min; frozen gloves: 90 min.
Simsek et al. [21] (2021), Turkey	Asia-Pacific Journal of Oncology Nursing	Multicenter three-arm parallel randomized clinical trial	*n* = 90 Breast cancer	Taxane	To compare the effect of cold application and exercise on the development of peripheral neuropathy in people with breast cancer and chemotherapy.	Exercise significantly reduces neuropathy symptoms of numbness in both hands and feet compared to cold application. The exercise program is more effective than cold application for preventing neuropathy in people receiving taxanes.	*Assessment* CIPNAT *Therapy*: Intervention Cold application vs. Exercise Cold application: Temperature −20/−30 °C. Time: 15 min before and during treatment, continuing for 24 h. Exercise: strengthening, stretching, and balance. Time: 15–30 min, 5/week.
Müller et al. [22] (2021), Germany	British Journal of Cancer	Intervention trial	*n* = 170 Breast (74%), pancreatic (6%), prostate (3%) and other cancers (17%)	Taxane, taxane-platinum, platinum	To investigate the preventive potential of sensorimotor and resistance training for peripheral neuropathy.	Sensorimotor and/or resistance training alleviate subjectively perceived sensory symptoms in the feet. Other clinically relevant results related to cancer therapy are achieved if an adequate training stimulus is achieved.	*Assessment* TNS, EORTC QLQ-CIPN15, EORTC QLQ-C30 *Therapy*: Preventive Sensorimotor and resistance exercise Program: sensorimotor 3/week for 35 min. Resistance 2/week for 45 min.
Oneda et al. [23] (2020), Italy	Integrative Cancer Therapies	Single-arm, single-center clinical pilot trial	*n* = 64 Breast (65.6%), gynecologic (31.3%), and pancreatic (3.1%) cancer	Taxane, platinum	To evaluate the prevention and reduction of peripheral neuropathy through the constant application of cold sleeves on the hands and feet.	Hilotherapy presents adequate efficacy and tolerability. This research shows that it seems to be able to prevent or reduce the symptoms of neuropathy. It is necessary to expand the study sample and add other treatment arms to the trial.	*Assessment* EORTC QLQ-C30, CTCAE *Therapy*: Preventive Hilotherm Device Temperature: 10 °C. Time: 30 min before and one hour after treatment.
Hirose, et al. [24] (2020), Japan	Supportive Care in Cancer	Descriptive single-center study	*n* = 4695 Colorectal (16.8%), breast (11.5%) cancer, and others	Taxane platinum	To investigate the relationship between chemotherapy-induced adverse events and QoL.	Peripheral neuropathy, malaise, extremity edema, and dry skin are significantly correlated with decreased QoL, regardless of the type of cancer or anticancer drugs used.	*Assessment* EuroQoL 5 Dimensión 5 Level (EQ-5D-5L)
Ruddy et al. [25] (2019), United States	Breast	Randomized prospective clinical pilot trial	*n* = 42 Breast cancer	Taxane	To assess the cooling of hands and feet during paclitaxel treatment to prevent peripheral neuropathy.	There were no significant differences in peripheral neuropathy between the group that received cryotherapy and the group that received control care. More research is needed, as the group that received cryotherapy had less neuropathy.	*Assessment* EORTC QLQ CIPN-20 *Therapy*: Preventive Cryotherapy Temperature: unspecified. Time: 15 min before and after treatment.
Griffiths et al. [26] (2018), United States	Supportive Care in Cancer	Cases and controls	*n* = 29 Breast cancer	Antracycline plus taxane	To assess the efficacy of cryotherapy in preventing taxane-induced neuropathic pain.	There were no significant differences in peripheral neuropathy between treated and untreated hands or feet. Safe and effective preventive strategies for peripheral neuropathy pain in oncology centers should be implemented.	*Assessment* BPI, QST, NPSI *Therapy*: Preventive Cold therapy Temperature: −25 to −30 °C. Time: 15 min prior and after paclitaxel and throughout 180 min infusion.
Kleckner et al. [27] (2018), United States	Supportive Care in Cancer	Multicenter randomized controlled clinical trial	*n* = 355 Breast (79%), lymphoma (5%), colon (5%), lung (3%), and other (7%) cancers	Taxane, platinum, taxane-platinum	To examine the effects of exercise on the symptoms of CIPN.	Exercise appears to reduce the symptoms of CIPN in patients receiving taxane, platinum, or alkaloid-based chemotherapy. Health professionals are encouraged to prescribe exercise for these patients.	*Assessment* CIPN Symptoms *Therapy*: Preventive Walking and resistance exercise Program: daily, six weeks.
Bao et al. [28] (2016), United States	Breast Cancer Research and Treatment	Longitudinal prospective study	*n* = 296 Breast cancer	Taxane	To determine the prevalence of CIPN, risk factors, and their association with long-term psychological distress and falls.	Of all studied patients, 58.4% reported neuropathic symptoms. People with neuropathy have greater psychological distress and numbers of falls. Obesity is a significant risk factor. Interventions should focus on incorporating fall prevention strategies and psycho-emotional interventions.	*Assessment* Neuropathic symptoms
Pachman et al. [29] (2015), United States	Supportive Care in Cancer	Open pilot trial	*n* = 37 Cancer types: 13 breast, 7 ovarian, 6 colon, and others	Taxane, platinum, taxane-platinum	To investigate the effect of Scrambler therapy for the treatment of CIPN.	Scrambler therapy using non-invasive cutaneous electrostimulation can be effective for the treatment of neuropathy. More research and placebo-controlled clinical trials with larger sample sizes are needed to assess its efficacy better.	*Assessment* Peripheral neuropathy symptom questionnaire *Therapy*: Intervention Scrambler therapy Program: daily sessions, ten consecutive days, 30 min.
Bland et al. [30] (2019), Canada	Clinical Breast Cancer	Randomized controlled trial	*n* = 27 Breast cancer	Taxane	To assess the effect of exercise on taxane-induced peripheral neuropathy in women with breast cancer.	Exercise may attenuate CIPN during the course of taxane chemotherapy and possibly improve adherence to taxanes in women with breast cancer. These findings need to be confirmed in more extensive trials.	*Assessment* EORTC QLQ CIPN20, Vibration Timing Test *Therapy*: Intervention Aerobic and resistance exercise. Program: 5 days/week.
Hanai et al. [31] (2018), Japan	Journal of the National Cancer Institute	Self-controlled clinical trial	*n* = 40 Breast cancer	Taxane	To assess the efficacy of cryotherapy in preventing neuropathy caused by chemotherapy.	Cryotherapy is useful in preventing both objective and subjective symptoms and dysfunction resulting from CIPN. The incidence of objective and subjective signs was clinically and statistically significantly lower in the intervention group than in the control group, being a simple and effective preventive strategy in the case of paclitaxel.	*Assessment* Tactile, Thermosensory, Vibration Disturbance and Patient-Reported Assessment *Therapy*: Prevent Cryotherapy Temperature: unspecified. Time: 15 min before to 15 min after treatment.

BPI, Brief Pain Inventory; CIPN, Chemotherapy Induced Peripheral Neuropathy; CIPNAT, Chemotherapy-Induced Peripheral Neuropathy Assessment Tool; EORTC QLQ-C30, European Organization for Research and Treatment of Cancer Quality of Life Questionnaire Core 30; EORTC QLQ CIPN, European Organization for Research and Treatment of Cancer Quality of Life-Chemotherapy Induced Peripheral Neuropathy Questionnaire; EQ-5D-5L, EuroQol 5 Dimension 5 Level; FACT/GOG-NtxS, Functional Assessment of Cancer Therapy/Gynecologic Oncology Group-Neurotoxicity subscale; NCI-CTCAE, Common Terminology Criteria for Adverse Events; NPSI, Neuropathic Pain Symptom Inventory; QoL: Quality of Life; TNSc, Total Neuropathy Score-Clinical Version.

## Data Availability

Not applicable.
